# 2′-Hy­droxy­methyl-1′-(4-methyl­phen­yl)-2′-nitro-1′,2′,5′,6′,7′,7a′-hexa­hydro­spiro­[indoline-3,3′-pyrrolizin]-2-one

**DOI:** 10.1107/S1600536811055449

**Published:** 2012-01-07

**Authors:** S. Sathya, Sundari Bhaskaran, G. Usha, N. Sivakumar, M. Bakthadoss

**Affiliations:** a Department of Physics, Queen Mary’s College, Chennai-4, Tamilnadu, India; b Department of Organic Chemistry, University of Madras, Guindy Campus, Chennai-25, Tamilnadu, India

## Abstract

In the title compound, C_22_H_23_N_3_O_4_, the tolyl ring is almost perpendicular [83.86 (7)°] to the best plane through the eight atoms of the pyrrolizidine ring system. The mol­ecular conformation is stabilized by an intra­molecular O—H⋯O hydrogen bond. The crystal packing features inversion dimers with *R*_2_^2^(8) motifs linked by pairs of N—H⋯O hydrogen bonds.

## Related literature

For indole derivatives, see: Ali *et al.* (1989 ▶); Nigović *et al.* (2000 ▶); Okabe & Adachi (1998 ▶); Oxford (1995 ▶); Schollmeyer *et al.* (1995 ▶); Taylor *et al.* (1999 ▶). For a related structure, see: Usha *et al.* (2005 ▶). For ring conformations, see Nardelli (1983 ▶).
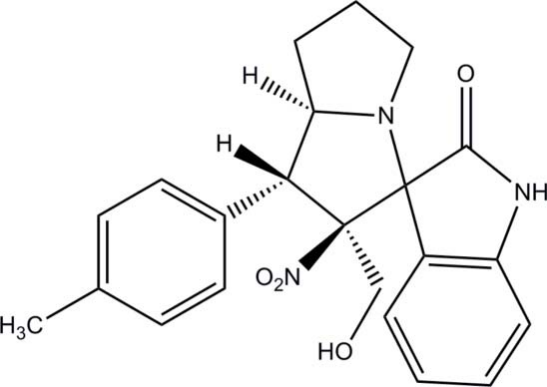


## Experimental

### 

#### Crystal data

C_22_H_23_N_3_O_4_
*M* *_r_* = 393.43
Triclinic, 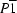

*a* = 8.9172 (4) Å
*b* = 9.9953 (4) Å
*c* = 11.5931 (6) Å
α = 81.257 (3)°
β = 76.638 (3)°
γ = 83.805 (2)°
*V* = 990.79 (8) Å^3^
*Z* = 2
Mo *K*α radiation
μ = 0.09 mm^−1^
*T* = 293 K
0.20 × 0.18 × 0.18 mm

#### Data collection

Bruker Kappa APEXII CCD diffractometer
18094 measured reflections
4912 independent reflections
3737 reflections with *I* > 2σ(*I*)
*R*_int_ = 0.028

#### Refinement

*R*[*F* ^2^ > 2σ(*F* ^2^)] = 0.048
*wR*(*F* ^2^) = 0.175
*S* = 1.25
4912 reflections
264 parameters
H-atom parameters constrained
Δρ_max_ = 0.50 e Å^−3^
Δρ_min_ = −0.34 e Å^−3^

### 

Data collection: *APEX2* (Bruker, 2004 ▶); cell refinement: *SAINT* (Bruker, 2004 ▶); data reduction: *SAINT*; program(s) used to solve structure: *SHELXS97* (Sheldrick, 2008 ▶); program(s) used to refine structure: *SHELXL97* (Sheldrick, 2008 ▶); molecular graphics: *ORTEP-3 for Windows* (Farrugia, 1997 ▶); software used to prepare material for publication: *SHELXL97* and *PLATON* (Spek, 2009 ▶).

## Supplementary Material

Crystal structure: contains datablock(s) I, global. DOI: 10.1107/S1600536811055449/bt5744sup1.cif

Structure factors: contains datablock(s) I. DOI: 10.1107/S1600536811055449/bt5744Isup2.hkl

Additional supplementary materials:  crystallographic information; 3D view; checkCIF report

## Figures and Tables

**Table 1:** Hydrogen-bond geometry (Å, °)

*D*—H⋯*A*	*D*—H	H⋯*A*	*D*⋯*A*	*D*—H⋯*A*
N1—H1*A*⋯O4^i^	0.86	2.00	2.8401 (15)	167
O3—H3*A*⋯O4	0.82	2.23	2.8686 (15)	135

Symmetry code: (i) 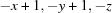.

**Fig. 1.**
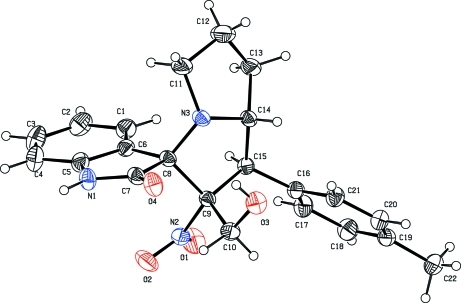
The molecular structure of the title compound, with displacement ellipsoids drawn at the 30% probability level.

**Fig. 2.**
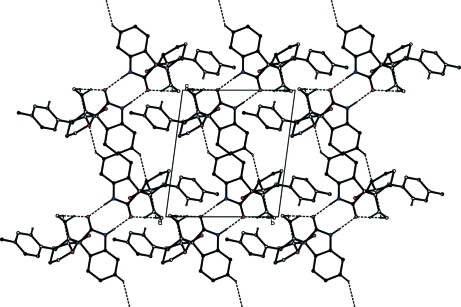
The packing of the molecules in the crystal structure. The dashed lines indicate the hydrogen bonds.

## supplementary crystallographic information

### Comment

The indole unit is observed in plants (Nigović *et al.*, 2000). Some of the indole derivatives exhibit anti-tumour (Schollmeyer *et al.*, 1995) and anti-bacterial (Okabe & Adachi,1998) activities. Sumatriptan, an indole derivative has been introduced into medicine as a drug for the treatment of migraine (Oxford, 1995). Pyrido [1, 2 - a]indole derivatives have been observed as potent inhibitors of HIV-typeI (Taylor *et al.*, 1999). Spiro-indoles have been reported to exhibit fungicidal activity(Ali *et al.*, 1989). In view of the wide spectrum of biological activity of indole and pyrrolizidine derivatives, the X-ray analysis of the title compound has been undertaken and the crystallographic details are reported in this communication. Bond lengths and bond angles of the pyrrolizidine group and the indole unit are in the same range as observed in reported structures (Usha *et al.*, 2005) The C7 = O4 double bond is slightly elongated [1.225 (2) Å] and this may be due to the active involvement of O4 both in intra and intermolecular hydrogen bonding. The pyrrolizidine ring is perpendicular to the methyl benzyl ring [89.8 (1)°]. The pyrrolizidine and methyl benzyl ring make angles of 81.7 (1) and 68.9 (1)°, respectively, with the oxindole system. The sum of the angles around N2 and N3 [360 and 341.7°], indicates *sp*^2^ and *sp*^3^ hybridization, respectively. The N3/C11—C14 ring adopts a half chair conformation with the smallest asymmetry parameter (Nardelli, 1983) of D2 (N3) = 0.018 (1)°. The asymmetry parameter DS (C9)= 0.024 (1)° indicates the half chair conformation of the N3/C8/C9/C15/C14 ring. The overall conformation of the pyrrolizidine ring is folded about the bridging bond N3—C14. In the title compound, each molecule is linked to two of its adjacent centro-symmetrically related molecules through N—H···O and C—H···O hydrogen bonds, forming dimers described by *R*^2^_2_(8) and *R*^2^_2_(18) rings. The atom O3 acts as a donor for an intrammolecular hydrogen bond.

### Experimental

A mixture of (E)-2- nitro-3-*p*-tolylprop-2-en-1-ol(2 mmol, 0.39 g),isatin(2 mmol, 0.29 g)and l-proline (2 mmol, 0.23 g) in acetonitrile(8 ml) was refluxed for 2 h. After the completion of the reaction as indicated by TLC, the reaction mixture was concentrated and the resulting crude mass was diluted with water (20 ml) and extracted with ethyl acetate (3x10ml) and dried over anhydrous NaSO4 The organic layer was concentrated and purified by column chromatography on silica gel (Acme 100–200 mesh), usingethyl acetate: hexanes (3:7) to provide as a colorless solid in 59% (0.46 g)yield.

### Refinement

H atoms were positioned geometrically and treated as riding on their parent atoms, with C—H = 0.93 - 0.97 Å, N– H = 0.86 Å, and O—H = 0.82Å and *U*_iso_(H) = 1.2*U*_eq_(N,O,C).

### Crystal data

**Table:**

C_22_H_23_N_3_O_4_	*Z* = 2
*M**_r_* = 393.43	*F*(000) = 416
Triclinic, *P*1	*D*_x_ = 1.319 Mg m^−^^3^
Hall symbol: -P 1	Mo *K*α radiation, λ = 0.71073 Å
*a* = 8.9172 (4) Å	Cell parameters from 4951 reflections
*b* = 9.9953 (4) Å	θ = 1.8–28.3°
*c* = 11.5931 (6) Å	µ = 0.09 mm^−^^1^
α = 81.257 (3)°	*T* = 293 K
β = 76.638 (3)°	Block, colorless
γ = 83.805 (2)°	0.20 × 0.18 × 0.18 mm
*V* = 990.79 (8) Å^3^	

### Data collection

**Table:**

Bruker Kappa APEXII CCD diffractometer	3737 reflections with *I* > 2σ(*I*)
Radiation source: fine-focus sealed tube	*R*_int_ = 0.028
graphite	θ_max_ = 28.4°, θ_min_ = 1.8°
ω and φ scan	*h* = −11→11
18094 measured reflections	*k* = −13→13
4912 independent reflections	*l* = −15→11

### Refinement

**Table:**

Refinement on *F*^2^	Primary atom site location: structure-invariant direct methods
Least-squares matrix: full	Secondary atom site location: difference Fourier map
*R*[*F*^2^ > 2σ(*F*^2^)] = 0.048	Hydrogen site location: inferred from neighbouring sites
*wR*(*F*^2^) = 0.175	H-atom parameters constrained
*S* = 1.25	*w* = 1/[σ^2^(*F*_o_^2^) + (0.1*P*)^2^] where *P* = (*F*_o_^2^ + 2*F*_c_^2^)/3
4912 reflections	(Δ/σ)_max_ = 0.007
264 parameters	Δρ_max_ = 0.50 e Å^−^^3^
0 restraints	Δρ_min_ = −0.34 e Å^−^^3^

### Special details

**Table:**

Geometry. All e.s.d.'s (except the e.s.d. in the dihedral angle between two l.s. planes) are estimated using the full covariance matrix. The cell e.s.d.'s are taken into account individually in the estimation of e.s.d.'s in distances, angles and torsion angles; correlations between e.s.d.'s in cell parameters are only used when they are defined by crystal symmetry. An approximate (isotropic) treatment of cell e.s.d.'s is used for estimating e.s.d.'s involving l.s. planes.
Refinement. Refinement of *F*^2^ against ALL reflections. The weighted *R*-factor *wR* and goodness of fit *S* are based on *F*^2^, conventional *R*-factors *R* are based on *F*, with *F* set to zero for negative *F*^2^. The threshold expression of *F*^2^ > σ(*F*^2^) is used only for calculating *R*-factors(gt) *etc*. and is not relevant to the choice of reflections for refinement. *R*-factors based on *F*^2^ are statistically about twice as large as those based on *F*, and *R*- factors based on ALL data will be even larger.

### Fractional atomic coordinates and isotropic or equivalent isotropic displacement parameters (Å^2^)

**Table:**

	*x*	*y*	*z*	*U*_iso_*/*U*_eq_	
C1	0.21898 (19)	0.62617 (16)	0.42758 (14)	0.0478 (4)	
H1	0.1948	0.7087	0.4579	0.057*	
C2	0.1894 (2)	0.50533 (18)	0.50230 (16)	0.0597 (5)	
H2	0.1468	0.5068	0.5833	0.072*	
C3	0.2229 (2)	0.38237 (18)	0.45695 (18)	0.0665 (5)	
H3	0.2010	0.3023	0.5080	0.080*	
C4	0.2878 (2)	0.37652 (16)	0.33808 (17)	0.0606 (5)	
H4	0.3103	0.2940	0.3076	0.073*	
C5	0.31830 (18)	0.49813 (14)	0.26526 (14)	0.0431 (3)	
C6	0.28473 (16)	0.62292 (13)	0.30774 (13)	0.0371 (3)	
C7	0.40581 (16)	0.64665 (13)	0.10119 (13)	0.0378 (3)	
C8	0.33024 (15)	0.73416 (12)	0.20230 (12)	0.0329 (3)	
C9	0.19143 (14)	0.83335 (12)	0.17272 (12)	0.0323 (3)	
C10	0.20643 (18)	0.88938 (15)	0.03935 (13)	0.0426 (3)	
H10A	0.2125	0.8142	−0.0059	0.051*	
H10B	0.1143	0.9472	0.0299	0.051*	
C11	0.56071 (19)	0.78765 (17)	0.27409 (19)	0.0574 (5)	
H11A	0.6583	0.7713	0.2186	0.069*	
H11B	0.5377	0.7054	0.3290	0.069*	
C12	0.5667 (2)	0.9037 (2)	0.3407 (2)	0.0771 (7)	
H12A	0.6405	0.9662	0.2935	0.093*	
H12B	0.5966	0.8709	0.4159	0.093*	
C13	0.40563 (19)	0.97253 (16)	0.36233 (15)	0.0511 (4)	
H13A	0.4066	1.0672	0.3718	0.061*	
H13B	0.3392	0.9272	0.4325	0.061*	
C14	0.35435 (15)	0.95803 (13)	0.24826 (13)	0.0360 (3)	
H14	0.3882	1.0341	0.1870	0.043*	
C15	0.18254 (15)	0.94197 (12)	0.25671 (12)	0.0328 (3)	
H15	0.1407	0.8995	0.3382	0.039*	
C16	0.08246 (15)	1.07192 (13)	0.23587 (12)	0.0365 (3)	
C17	−0.06238 (17)	1.08928 (15)	0.31073 (15)	0.0471 (4)	
H17	−0.0947	1.0221	0.3739	0.056*	
C18	−0.15936 (19)	1.20451 (16)	0.29307 (16)	0.0521 (4)	
H18	−0.2560	1.2128	0.3442	0.063*	
C19	−0.11602 (18)	1.30755 (15)	0.20124 (15)	0.0473 (4)	
C20	0.0293 (2)	1.29239 (15)	0.12843 (15)	0.0497 (4)	
H20	0.0624	1.3614	0.0672	0.060*	
C21	0.12749 (18)	1.17662 (14)	0.14433 (14)	0.0443 (3)	
H21	0.2243	1.1690	0.0933	0.053*	
C22	−0.2232 (2)	1.42970 (17)	0.17871 (19)	0.0633 (5)	
H22A	−0.3157	1.4262	0.2406	0.095*	
H22B	−0.2490	1.4310	0.1026	0.095*	
H22C	−0.1735	1.5104	0.1785	0.095*	
N1	0.38738 (15)	0.51498 (12)	0.14359 (12)	0.0456 (3)	
H1A	0.4148	0.4495	0.1010	0.055*	
N2	0.04800 (14)	0.75474 (12)	0.20397 (12)	0.0422 (3)	
N3	0.43621 (12)	0.83081 (11)	0.20981 (11)	0.0381 (3)	
O1	−0.06118 (13)	0.78766 (13)	0.28004 (13)	0.0675 (4)	
O2	0.05119 (15)	0.65971 (13)	0.14818 (13)	0.0665 (4)	
O3	0.33650 (13)	0.96383 (11)	−0.00794 (10)	0.0511 (3)	
H3A	0.4152	0.9135	−0.0065	0.077*	
O4	0.47420 (14)	0.69055 (10)	0.00021 (10)	0.0502 (3)	

### Atomic displacement parameters (Å^2^)

**Table:**

	*U*^11^	*U*^22^	*U*^33^	*U*^12^	*U*^13^	*U*^23^
C1	0.0539 (9)	0.0497 (8)	0.0399 (9)	0.0035 (7)	−0.0110 (7)	−0.0100 (6)
C2	0.0661 (11)	0.0658 (11)	0.0403 (9)	0.0023 (9)	−0.0074 (8)	0.0034 (8)
C3	0.0788 (13)	0.0494 (9)	0.0619 (12)	0.0034 (8)	−0.0128 (10)	0.0120 (8)
C4	0.0760 (13)	0.0385 (8)	0.0622 (12)	0.0062 (8)	−0.0124 (10)	−0.0019 (7)
C5	0.0454 (8)	0.0383 (7)	0.0451 (9)	0.0042 (6)	−0.0102 (7)	−0.0086 (6)
C6	0.0384 (7)	0.0374 (6)	0.0366 (7)	0.0021 (5)	−0.0115 (6)	−0.0066 (5)
C7	0.0365 (7)	0.0376 (7)	0.0417 (8)	−0.0003 (5)	−0.0070 (6)	−0.0164 (6)
C8	0.0322 (7)	0.0335 (6)	0.0348 (7)	−0.0006 (5)	−0.0067 (5)	−0.0127 (5)
C9	0.0279 (6)	0.0335 (6)	0.0367 (7)	−0.0031 (5)	−0.0075 (5)	−0.0077 (5)
C10	0.0473 (8)	0.0464 (8)	0.0363 (8)	−0.0036 (6)	−0.0115 (6)	−0.0086 (6)
C11	0.0405 (8)	0.0548 (9)	0.0891 (13)	0.0092 (7)	−0.0314 (9)	−0.0296 (9)
C12	0.0755 (13)	0.0767 (12)	0.1030 (17)	0.0189 (10)	−0.0583 (13)	−0.0437 (12)
C13	0.0588 (10)	0.0488 (8)	0.0568 (10)	0.0035 (7)	−0.0270 (8)	−0.0244 (7)
C14	0.0352 (7)	0.0335 (6)	0.0431 (8)	0.0002 (5)	−0.0114 (6)	−0.0143 (5)
C15	0.0317 (7)	0.0334 (6)	0.0328 (7)	0.0009 (5)	−0.0051 (5)	−0.0083 (5)
C16	0.0352 (7)	0.0366 (6)	0.0387 (8)	0.0021 (5)	−0.0083 (6)	−0.0114 (5)
C17	0.0423 (8)	0.0444 (8)	0.0492 (9)	0.0032 (6)	−0.0003 (7)	−0.0094 (6)
C18	0.0387 (8)	0.0524 (9)	0.0638 (11)	0.0091 (7)	−0.0055 (7)	−0.0207 (8)
C19	0.0476 (9)	0.0398 (7)	0.0627 (10)	0.0066 (6)	−0.0251 (8)	−0.0186 (7)
C20	0.0558 (10)	0.0395 (7)	0.0556 (10)	−0.0003 (7)	−0.0198 (8)	−0.0029 (7)
C21	0.0405 (8)	0.0410 (7)	0.0489 (9)	0.0015 (6)	−0.0066 (7)	−0.0062 (6)
C22	0.0604 (11)	0.0472 (9)	0.0901 (14)	0.0132 (8)	−0.0346 (10)	−0.0176 (9)
N1	0.0561 (8)	0.0340 (6)	0.0454 (7)	0.0031 (5)	−0.0045 (6)	−0.0154 (5)
N2	0.0355 (6)	0.0417 (6)	0.0514 (8)	−0.0069 (5)	−0.0139 (6)	−0.0031 (5)
N3	0.0280 (6)	0.0383 (6)	0.0525 (7)	0.0012 (4)	−0.0111 (5)	−0.0192 (5)
O1	0.0370 (6)	0.0687 (8)	0.0894 (10)	−0.0126 (6)	0.0076 (6)	−0.0141 (7)
O2	0.0668 (8)	0.0596 (7)	0.0847 (10)	−0.0225 (6)	−0.0224 (7)	−0.0237 (7)
O3	0.0543 (7)	0.0523 (6)	0.0417 (6)	−0.0083 (5)	−0.0003 (5)	−0.0035 (5)
O4	0.0595 (7)	0.0436 (6)	0.0429 (6)	−0.0035 (5)	0.0053 (5)	−0.0169 (5)

### Geometric parameters (Å, °)

**Table:**

C1—C6	1.381 (2)	C12—H12A	0.9700
C1—C2	1.387 (2)	C12—H12B	0.9700
C1—H1	0.9300	C13—C14	1.527 (2)
C2—C3	1.386 (3)	C13—H13A	0.9700
C2—H2	0.9300	C13—H13B	0.9700
C3—C4	1.373 (3)	C14—N3	1.4732 (16)
C3—H3	0.9300	C14—C15	1.5367 (18)
C4—C5	1.385 (2)	C14—H14	0.9800
C4—H4	0.9300	C15—C16	1.5153 (17)
C5—C6	1.3878 (19)	C15—H15	0.9800
C5—N1	1.3950 (19)	C16—C17	1.390 (2)
C6—C8	1.5331 (19)	C16—C21	1.392 (2)
C7—O4	1.2274 (18)	C17—C18	1.383 (2)
C7—N1	1.3465 (18)	C17—H17	0.9300
C7—C8	1.5532 (18)	C18—C19	1.381 (2)
C8—N3	1.4472 (16)	C18—H18	0.9300
C8—C9	1.5686 (18)	C19—C20	1.381 (2)
C9—N2	1.5167 (16)	C19—C22	1.496 (2)
C9—C10	1.543 (2)	C20—C21	1.389 (2)
C9—C15	1.5493 (17)	C20—H20	0.9300
C10—O3	1.4028 (18)	C21—H21	0.9300
C10—H10A	0.9700	C22—H22A	0.9600
C10—H10B	0.9700	C22—H22B	0.9600
C11—N3	1.4680 (19)	C22—H22C	0.9600
C11—C12	1.499 (2)	N1—H1A	0.8600
C11—H11A	0.9700	N2—O1	1.2067 (18)
C11—H11B	0.9700	N2—O2	1.2223 (17)
C12—C13	1.507 (2)	O3—H3A	0.8200
			
C6—C1—C2	119.44 (15)	C12—C13—H13A	111.4
C6—C1—H1	120.3	C14—C13—H13A	111.4
C2—C1—H1	120.3	C12—C13—H13B	111.4
C1—C2—C3	120.40 (17)	C14—C13—H13B	111.4
C1—C2—H2	119.8	H13A—C13—H13B	109.2
C3—C2—H2	119.8	N3—C14—C13	104.96 (11)
C4—C3—C2	121.25 (16)	N3—C14—C15	105.21 (10)
C4—C3—H3	119.4	C13—C14—C15	118.35 (12)
C2—C3—H3	119.4	N3—C14—H14	109.3
C3—C4—C5	117.47 (16)	C13—C14—H14	109.3
C3—C4—H4	121.3	C15—C14—H14	109.3
C5—C4—H4	121.3	C16—C15—C14	116.15 (11)
C4—C5—C6	122.65 (15)	C16—C15—C9	117.55 (11)
C4—C5—N1	126.82 (14)	C14—C15—C9	101.87 (10)
C6—C5—N1	110.52 (12)	C16—C15—H15	106.8
C1—C6—C5	118.79 (14)	C14—C15—H15	106.8
C1—C6—C8	133.01 (12)	C9—C15—H15	106.8
C5—C6—C8	108.19 (12)	C17—C16—C21	117.36 (13)
O4—C7—N1	125.80 (13)	C17—C16—C15	119.24 (13)
O4—C7—C8	125.37 (12)	C21—C16—C15	123.40 (12)
N1—C7—C8	108.81 (12)	C18—C17—C16	121.25 (15)
N3—C8—C6	119.23 (11)	C18—C17—H17	119.4
N3—C8—C7	109.64 (10)	C16—C17—H17	119.4
C6—C8—C7	100.66 (10)	C17—C18—C19	121.52 (15)
N3—C8—C9	100.32 (9)	C17—C18—H18	119.2
C6—C8—C9	114.29 (11)	C19—C18—H18	119.2
C7—C8—C9	113.20 (10)	C18—C19—C20	117.47 (13)
N2—C9—C10	104.26 (11)	C18—C19—C22	121.66 (15)
N2—C9—C15	112.33 (11)	C20—C19—C22	120.85 (16)
C10—C9—C15	115.03 (11)	C19—C20—C21	121.66 (15)
N2—C9—C8	108.15 (10)	C19—C20—H20	119.2
C10—C9—C8	115.16 (11)	C21—C20—H20	119.2
C15—C9—C8	101.99 (10)	C20—C21—C16	120.71 (14)
O3—C10—C9	113.06 (12)	C20—C21—H21	119.6
O3—C10—H10A	109.0	C16—C21—H21	119.6
C9—C10—H10A	109.0	C19—C22—H22A	109.5
O3—C10—H10B	109.0	C19—C22—H22B	109.5
C9—C10—H10B	109.0	H22A—C22—H22B	109.5
H10A—C10—H10B	107.8	C19—C22—H22C	109.5
N3—C11—C12	104.69 (13)	H22A—C22—H22C	109.5
N3—C11—H11A	110.8	H22B—C22—H22C	109.5
C12—C11—H11A	110.8	C7—N1—C5	111.50 (12)
N3—C11—H11B	110.8	C7—N1—H1A	124.3
C12—C11—H11B	110.8	C5—N1—H1A	124.2
H11A—C11—H11B	108.9	O1—N2—O2	123.69 (13)
C11—C12—C13	105.71 (14)	O1—N2—C9	119.96 (12)
C11—C12—H12A	110.6	O2—N2—C9	116.35 (12)
C13—C12—H12A	110.6	C8—N3—C11	119.86 (12)
C11—C12—H12B	110.6	C8—N3—C14	111.92 (10)
C13—C12—H12B	110.6	C11—N3—C14	109.93 (11)
H12A—C12—H12B	108.7	C10—O3—H3A	109.5
C12—C13—C14	102.09 (13)		
			
C6—C1—C2—C3	−1.1 (3)	N2—C9—C15—C16	−76.79 (15)
C1—C2—C3—C4	0.9 (3)	C10—C9—C15—C16	42.26 (16)
C2—C3—C4—C5	−0.1 (3)	C8—C9—C15—C16	167.64 (11)
C3—C4—C5—C6	−0.6 (3)	N2—C9—C15—C14	155.06 (11)
C3—C4—C5—N1	178.06 (17)	C10—C9—C15—C14	−85.88 (13)
C2—C1—C6—C5	0.5 (2)	C8—C9—C15—C14	39.49 (13)
C2—C1—C6—C8	179.21 (15)	C14—C15—C16—C17	−136.58 (14)
C4—C5—C6—C1	0.4 (2)	C9—C15—C16—C17	102.44 (15)
N1—C5—C6—C1	−178.47 (13)	C14—C15—C16—C21	43.66 (18)
C4—C5—C6—C8	−178.65 (15)	C9—C15—C16—C21	−77.31 (17)
N1—C5—C6—C8	2.51 (17)	C21—C16—C17—C18	1.6 (2)
C1—C6—C8—N3	56.6 (2)	C15—C16—C17—C18	−178.20 (13)
C5—C6—C8—N3	−124.58 (13)	C16—C17—C18—C19	−0.6 (2)
C1—C6—C8—C7	176.41 (15)	C17—C18—C19—C20	−1.0 (2)
C5—C6—C8—C7	−4.77 (14)	C17—C18—C19—C22	177.39 (15)
C1—C6—C8—C9	−61.95 (19)	C18—C19—C20—C21	1.7 (2)
C5—C6—C8—C9	116.88 (12)	C22—C19—C20—C21	−176.77 (14)
O4—C7—C8—N3	−46.30 (18)	C19—C20—C21—C16	−0.7 (2)
N1—C7—C8—N3	132.12 (12)	C17—C16—C21—C20	−0.9 (2)
O4—C7—C8—C6	−172.79 (14)	C15—C16—C21—C20	178.82 (13)
N1—C7—C8—C6	5.63 (14)	O4—C7—N1—C5	173.80 (14)
O4—C7—C8—C9	64.80 (18)	C8—C7—N1—C5	−4.61 (17)
N1—C7—C8—C9	−116.79 (13)	C4—C5—N1—C7	−177.40 (16)
N3—C8—C9—N2	−160.20 (10)	C6—C5—N1—C7	1.38 (19)
C6—C8—C9—N2	−31.39 (14)	C10—C9—N2—O1	−119.17 (15)
C7—C8—C9—N2	83.07 (13)	C15—C9—N2—O1	6.03 (18)
N3—C8—C9—C10	83.67 (12)	C8—C9—N2—O1	117.81 (14)
C6—C8—C9—C10	−147.53 (11)	C10—C9—N2—O2	60.59 (15)
C7—C8—C9—C10	−33.06 (15)	C15—C9—N2—O2	−174.22 (12)
N3—C8—C9—C15	−41.62 (12)	C8—C9—N2—O2	−62.43 (16)
C6—C8—C9—C15	87.19 (12)	C6—C8—N3—C11	34.10 (18)
C7—C8—C9—C15	−158.34 (10)	C7—C8—N3—C11	−81.02 (16)
N2—C9—C10—O3	−178.62 (11)	C9—C8—N3—C11	159.63 (13)
C15—C9—C10—O3	57.92 (16)	C6—C8—N3—C14	−96.79 (14)
C8—C9—C10—O3	−60.29 (15)	C7—C8—N3—C14	148.08 (11)
N3—C11—C12—C13	27.5 (2)	C9—C8—N3—C14	28.74 (14)
C11—C12—C13—C14	−35.8 (2)	C12—C11—N3—C8	−139.59 (16)
C12—C13—C14—N3	30.41 (17)	C12—C11—N3—C14	−7.8 (2)
C12—C13—C14—C15	147.33 (15)	C13—C14—N3—C8	121.37 (13)
N3—C14—C15—C16	−151.72 (11)	C15—C14—N3—C8	−4.22 (15)
C13—C14—C15—C16	91.49 (15)	C13—C14—N3—C11	−14.42 (16)
N3—C14—C15—C9	−22.68 (13)	C15—C14—N3—C11	−140.01 (13)
C13—C14—C15—C9	−139.47 (12)		

### Hydrogen-bond geometry (Å, °)

**Table:**

*D*—H···*A*	*D*—H	H···*A*	*D*···*A*	*D*—H···*A*
N1—H1A···O4^i^	0.86	2.00	2.8401 (15)	167.
O3—H3A···O4	0.82	2.23	2.8686 (15)	135.

Symmetry codes: (i) −*x*+1, −*y*+1, −*z*.
